# UFT/leucovorin and oxaliplatin alternated with UFT/leucovorin and irinotecan in metastatic colorectal cancer

**DOI:** 10.1038/sj.bjc.6601521

**Published:** 2004-01-20

**Authors:** R Petrioli, M Sabatino, A I Fiaschi, S Marsili, D Pozzessere, S Messinese, P Correale, S Civitelli, G Tanzini, F Tani, A De Martino, G Marzocca, M Lorenzi, G Giorgi, G Francini

**Affiliations:** 1 Department of Human Pathology and Oncology, Medical Oncology Section, University of Siena, Viale Bracci 11, Siena 53100, Italy; 2 Department of Pharmacology, University of Siena, Siena, Italy; 3 Clinical Surgery; University of Siena, Siena, Italy; 4 General Surgery; University of Siena, Siena, Italy

**Keywords:** colorectal cancer, irinotecan, oxaliplatin, tegafur/uracil

## Abstract

A total of 41 metastatic colorectal cancer (CRC) patients received tegafur/uracil (UFT)+leucovorin (LV)+oxaliplatin alternated with UFT/LV+irinotecan. The overall response rate was 58.5% (95% confidence interval, 42.2–73.3%), and the median progression-free survival was 8.8 months. There were no grade 4 toxicities; 12 patients (29%) experienced grade 3 diarrhoea. There were no cases of hand–foot syndrome. This alternating regimen seems to be effective and well tolerated in the first-line treatment of patients with metastatic CRC.

Recent developments in the treatment of colorectal cancer (CRC) include the introduction of oral fluoropyrimidines, such as tegafur/uracil (UFT) and capecitabine, which may replace infusional 5-FU (Cunningham and James, 2001). Tegafur/uracil is a fixed combination of tegafur (1-2(tetrahydrofuranyl)-5-fluorouracil) and uracil in a 1 : 4 molar ratio (Maehara *et al*, 1997): tegafur is an orally bioavailable prodrug of 5-FU, and uracil reversibly inhibits dihydropyrimidine dehydrogenase, the primary 5-FU catabolic enzyme.

After the promising response rates reported in phase II, two randomised trials of the UFT/leucovorin (LV) combination found that it was as efficacious as conventional 5-FU/LV, but had a better safety profile (Carmichael *et al*, 2002; Douillard *et al*, 2002). Furthermore, the results of phase I/II studies of UFT/LV+oxaliplatin (L-HOP) or UFT/LV+irinotecan (CPT-11) combinations indicate their favourable antitumour activity (Price and Hill, 2000; Alonso *et al*, 2001; Vanhoefer and Wilke, 2001).

In order to increase the efficacy while minimising toxicity, we designed a new chemotherapy protocol in which UFT/LV is alternately combined with L-HOP and CPT-11. The aim of this phase II study was to evaluate the antitumour activity and toxicity of this new regimen in patients with metastatic CRC.

## MATERIALS AND METHODS

### Eligibility criteria

The eligibility criteria were histologically proven metastatic colon or rectum adenocarcinoma, no previous chemotherapy for metastatic disease, age 18–75 years, an ECOG performance status of 0–2, bidimensionally measurable disease, a life expectancy of at least 3 months, an absolute neutrophil count of ⩾1.5 × 10^9^ l^−1^ and platelet count of ⩾100 × 10^9^ l^−1^, and creatinine and total bilirubin levels ⩽1.25 times the upper normal limit. Adjuvant 5-FU-based chemotherapy had to be completed >6 months before entry.

The exclusion criteria were operable metastatic disease, severe cardiac dysfunction, chronic diarrhoea or uncontrolled infection.

The study was approved by our local Ethics and Scientific Committee; all of the patients gave their written informed consent.

### Patient evaluation

The pretreatment evaluation included a detailed history and physical examination, complete blood cell count, whole blood chemistry, and chest and abdominal computed tomography (CT). During treatment, the patients underwent weekly complete blood cell counts, fortnightly clinical assessments, and routine biochemical tests.

Response was evaluated after two 35-day cycles (sooner if clinically indicated) using WHO criteria (Miller *et al*, 1981), and confirmed after 4 weeks by means of repeat CT scans. The results were reviewed by an independent Review Committee of three radiologists blinded to the investigator's measurements, whose assessments were exclusively based on imaging techniques.

Toxicity was assessed using the NCI criteria. Treatment was delayed if neutrophils were <1500 mm^−3^, platelets <1 00 000 mm^−3^, or the patient had persistent grade >1 diarrhoea or stomatitis.

UFT intake was interrupted for grade >2 nonhaematological toxicity, and not resumed until this was grade ⩽1. The dose was reduced by 50 mg m^−2^ day^−1^ in the treatment cycles following the appearance of grade 3 or 4 haematological or nonhaematological toxicities. If UFT was discontinued or doses were missed, LV was not given. The study medication returned by the patient was counted at each cycle.

After the appearance of grade ⩾3 nonhaematological or grade 4 haematological toxicity, the L-HOP or CPT-11 dose was reduced by 25% in subsequent cycles. L-HOP was also reduced by 25% for persistent (⩾14 days) paraesthesia or temporary (7–14 days) painful paraesthesia or functional impairment. In cases of persistent (⩾14 days) painful paraesthesia or functional impairment, L-HOP was omitted from subsequent cycles until recovery.

### Treatment

Oral UFT (250 mg m^−2^ day^−1^) and LV (90 mg day^−1^) were given for 28 days of a 35-day cycle, combined with a 2-h infusion of L-HOP (85 mg m^−2^) on days 1 and 15. On day 35, the next cycle consisted of oral UFT/LV at the same doses for 28 days, combined with a 90-min infusion of CPT-11 (180 mg m^−2^) on days 35 and 49. The treatments were alternated until disease progression, unacceptable toxicity or consent withdrawal.

The total daily UFT dose was divided into three administrations (rounded to the nearest 100 mg) every 8 h; if the doses could not be divided equally, the highest was administered in the morning.

Intravenous (i.v.) 5-hydroxytryptamine-3 antagonists plus dexamethasone 8 mg i.v. were administered before the infusions. No cytokine prophylaxis was recommended.

### Statistical analysis

As at least 40% of patients respond to standard 5-FU/LV+CPT-11 or 5-FU/LV+L-HOP combinations, a >60% response to a new regimen with acceptable toxicity can be considered promising. Using Simon's two-stage minimax design, with alpha and beta error probabilities of 0.10, at least 41 patients were required (Simon, 1989).

## RESULTS

A total of 41 patients with metastatic CRC were enrolled between September 2001 and January 2003 (Table 1

**Table 1:** Patient characteristics

**Characteristic**	**No. of patients (*n*=41)**	**%**
*Age (years)*		
Median	65	
Range	46–75	
		
*Sex*		
Male	23	56
Female	18	44
		
*ECOG performance status*		
0	27	66
1	10	24
2	4	10
		
*Primary tumour*		
Colon	34	83
Rectum	7	17
Previous primary tumour surgery	35	85
		
*Metastatic sites*		
Liver	36	88
Lung	7	17
Lymph nodes	8	20
Local abdominal mass	7	17
Peritoneum	4	10
Bone	1	2
Pleural effusion	1	2
		
*No. of metastatic sites*		
1	22	54
2	16	39
>3	3	7
Previous adjuvant chemotherapy	16	39
Previous adjuvant radiotherapy	3	7

). A total of 177 chemotherapeutic cycles were administered for a median of five cycles/patient (range 2–8). All of the patients were assessable for treatment response and toxicity.

Intent-to-treat analysis showed complete responses in three patients (7.3%) and partial responses in 21 (51.2%): an overall response rate of 58.5% (95% CI 42.2–73.3%) confirmed by an independent radiological review. In all, 13 patients (31.7%) had stable disease, and four (9.8%) progressive disease. The median response duration was 7.1 months (range 3.2–16.4). Postchemotherapy surgery of residual metastases was attempted in eight patients (19.5%) with liver involvement only, and was macroscopically complete in four (9.8%). After the study, 12 patients received second-line chemotherapy with mitomycin and six a weekly 5-FU/LV bolus.

**Figure 1:**
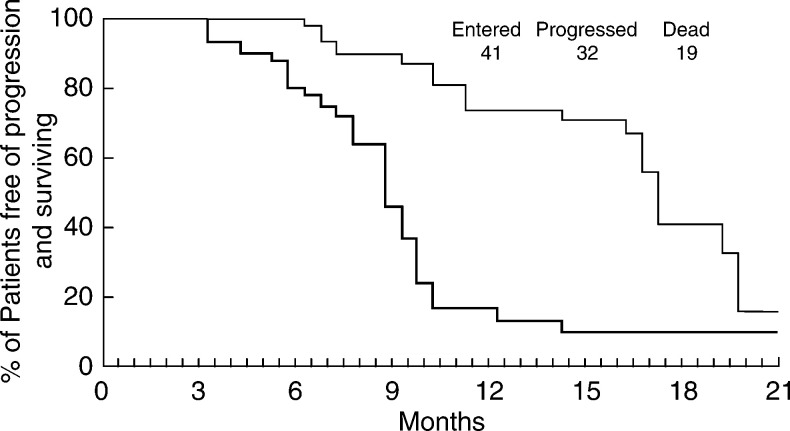
Kaplan–Meier curves for progression-free survival (−) and overall survival (−).

The median follow-up was 15.6 months; median progression-free survival (PFS) 8.8 months (95% CI: 7.4–10.2); median overall survival 17.3 months (95% CI: 14.6–20.4) (Figure 1

).

### Treatment toxicity

There were no grade 4 toxicities; grade 3 toxicity was uncommon except for diarrhoea (Table 2

**Table 2:** Maximum toxicity per patient (41 enrolled patients)

	**NCI grade (%)**			
	**1**	**2**	**3**	**4**
*Haematological toxicity*				
Neutropenia	59	22	5	—
Anaemia	20	10	2	—
Thrombocytopenia	17	7	2	—
				
*Nonhaematological toxicity*				
Nausea/vomiting	46	41	7	—
Diarrhoea	17	54	29	—
Stomatitis	24	7	—	—
Neurotoxicity	22	15	2	—
Hyperbilirubinaemia	29	5	2	—

). The patients were all treated as outpatients, and none was hospitalised because of adverse events. No cardiac or vascular toxicity was observed, and no cases of hand–foot syndrome. Three patients (7%) showed increased bilirubin levels (grade 2–3) unassociated with concurrent severely increased transaminases; all resumed treatment after recovery. During treatment, 6% of the cycles were associated with grade 3 diarrhoea, 3% with grade 3 neutropenia, and 2% with grade 3 nausea/vomiting. Diarrhoea peaked in cycle 2, and was reduced in the following cycles by dose adaptations.

### Drug exposure

The median received cumulative dose intensity was 32 g m^−2^ for the fluoropyrimidine, 470 mg m^−2^ for L-HOP, and 640 mg m^−2^ for CPT-11. The median received dose intensities during the first two cycles were, respectively, 1372 (98% of planned), 16.6 (98%), and 34.9 mg m^−2^ week^−1^ (97%).

In all, 12 patients (29%) and 21 cycles (12%) required dose reductions of at least one drug. A total of 34 cycles (19%) were delayed by >1 week because of diarrhoea (9%), neutropenia (2%), other toxicities (2%), or reasons unrelated to the treatment (6%). Tegafur/uracil was interrupted for a median of 3 days (range: 1–7) in 33% of the cycles, because of diarrhoea or other toxicities.

## DISCUSSION

Our results indicate the feasibility and efficacy of UFT/LV+L-HOP alternated with UFT/LV+CPT-11 in non-pretreated metastatic CRC patients. This regimen predictably meant low dose intensities of L-HOP (planned 17 mg m^−2^ week^−1^) and CPT-11 (planned 36 mg m^−2^ week^−1^), but our results (58.5% response rate) and other recent clinical data suggest that prolonged tumour exposure to a fluoropyrimidine plus full doses of L-HOP alternated with full doses of CPT-11 can be highly efficacious in metastatic CRC (Aschele *et al*, 2002; Aparicio *et al*, 2003). Comparison of this small phase II study with similar experiences including other oral drugs such as capecitabine is difficult, above all because of their different schedules. A fixed UFT dose of 250 mg m^−2^ day^−1^ for 28 days was chosen on the basis of the proved activity and tolerability of continuous administration and the results of previous phase I/II studies (Twelves, 1999; Price and Hill, 2000; Vanhoefer and Wilke, 2001). A biweekly schedule was chosen for CPT-11 or L-HOP because it is active and has a favourable toxicity profile (de Gramont *et al*, 2000; Douillard *et al*, 2000).

More than 50% of our patients were chemonaive, only four had peritoneal disease, and 66% had an ECOG performance status of 0, thus indicating a better than average group with regard to efficacy and toxicity. Furthermore, the CR rate remained low (7.3%) and, if an increase in the rate of CR or PFS cannot be achieved by combining all the three active compounds in one regimen, much can be said in favour of sequential treatment regimens.

Our alternating regimen was well tolerated as the proportion of patients with grade 3 diarrhoea (29%) was only slightly higher than that reported with UFT/LV alone. Grade 3 diarrhoea was most frequent during cycle 2, which suggests a cumulative component and/or overlapping toxicity with CPT-11.

The low level of haematological toxicity was mainly due to the choice of an alternating regimen, which favours safety (no overlapping toxicity) at the expense of dose intensity. Nevertheless, a high incidence of severe neutropenia, associated with greater use of haematopoietic growth factors, is often reported when 5-FU, L-HOP, and CPT-11 are simultaneously combined (Falcone *et al*, 2002; Souglakos *et al*, 2002). Hand–foot syndrome, which is frequently described with the use of continuous 5-FU infusions or capecitabine (Van Cutsem *et al*, 2000), was never observed, possibly because the profile of frequent oral UFT/LV dosing may resemble that of a repeated mini-bolus (Ho *et al*, 1998) and the UFT dose (250 m^−2^ day^−1^) was lower than the 300–350 mg m^−2^ day^−1^ used by other authors. The complete absence of hand–foot syndrome is very promising and a major advantage over similar studies of capecitabine. However, only a randomised trial can clarify the question as to whether capecitabine or UFT is the best oral 5-FU drug for combination chemotherapy.

The low level of neurotoxicity was due to the low median cumulative L-HOP dose per patient (470 mg m^−2^), and the fact that a biweekly schedule of a dose of 85 mg m^−2^ in a 2-h infusion may minimise the symptoms of chronic cumulative neuropathy (Cassidy and Misset, 2002).

In conclusion, our findings suggest that the combination of UFT/LV+L-HOP alternated with UFT/LV+CPT-11 is an effective and well-tolerated regimen for the first-line treatment of metastatic CRC.
